# A review and analysis of the business model innovation literature

**DOI:** 10.1016/j.heliyon.2023.e17895

**Published:** 2023-07-03

**Authors:** WenJun Huang, Takeyasu Ichikohji

**Affiliations:** Graduate School of Economics and Management, Tohoku University, Sendai, Japan

**Keywords:** Business model innovation, Systematic literature review, Theoretical framework, Research agenda

## Abstract

Business model innovation (BMI) is an emerging field that has attracted much attention from scholars and practitioners. However, the literature on BMI is fragmented and inconsistent, lacking a comprehensive and systematic framework. This study aims to fill this gap by conducting a literature review of 272 peer-reviewed articles on BMI published between 2010 and 2022. We analyze the articles based on six dimensions: antecedents, processes, types, barriers, outcomes, and moderators/mediators of BMI. We synthesize the findings and propose an integrated theoretical model that captures the complex relationships among these dimensions. We also identify several research gaps and directions for future studies. This study contributes to the BMI literature by providing a clearer understanding of this phenomenon and offering practical guidance for various types of businesses.

## 1 Introduction

Since the turn of the 21st century, the rapid development of information technology, economic integration, and globalization, are leading to a rapidly changing and uncertain competitive environment for companies [1]. Under such circumstances, many companies are resistant to spending money on product and service innovation, because of not only the cost but also uncertain returns. Furthermore, in highly competitive and ever-changing markets, new products and services are extremely susceptible to imitation in developing countries such as China and India. However, as companies continue to evolve, they are now increasingly turning to BMI (business model innovation) as a complement or alternative to product, service, and process innovation [2], not only because it enables the development of a clear logic rooted in organizational processes [3], but also because it allows the management to address the obvious trade-off between the costs and benefits of innovation [4].

BMI can theoretically help companies deal with homogeneity and uncertainty of returns, and in practice, there exist successful examples such as Google, Walmart, IBM, Netflix, and Amazon, which support this point, but many companies have not been successful in innovating their business models (BM) [[5], [6], [7]]. This is because BMI is a complex and multidimensional process involving multiple levels, stages, and elements [8,9].

Despite the rapid growth of research on the BMI phenomenon and the large number of theoretical and empirical studies generated during the last two to three decades, the current theoretical accumulation on BMI is still in a preliminary and confusing stage. This is because different studies have adopted different theoretical perspectives, levels of analysis, and scopes of research [10], resulting in controversy and inconsistency over the antecedents, processes, types, barriers, outcomes, and moderators/mediators of BMI [8]. In addition, existing reviews on BMI are limited and insufficient. Although some systematic literature reviews on BMI have been published so far in order to address the theoretical fragmentation and dispersion of BMI [10], these reviews have mainly focused on specific aspects of BMI, such as process [11], antecedent [12], or its antecedents, consequences, and outcomes [8,12,13] (See Table 1). None of these studies have provided an in-depth and extensive analysis of the six aspects of BMI: antecedents, processes, types, barriers, outcomes, and potential moderators/mediator factors. Therefore, a comprehensive and systematic framework that can integrate different perspectives and explain various aspects of BMI is needed.

**Table 1:** Review of previous literature on business model innovation.

Author	[17]	[8]	[18]	[4]	[20]	[12]	[11]
Research Direction	Antecedents, processes & elements and impacts	Concept, Process, Result, Consequence	Process	Internal factors & consequences	Components	Key components	Process
Period	1981–2012	2000–2015	2000–2014	2000–2017	2010–2016	2016–2019	2001–2020
Databases	ISI Web of Knowledge	EBSCO: Business source premier	EBSCO: Academic Search Complete; Business Source Complete	EBSCO; ProQuest; JSTOR; Scopus	EBSCO Business Complete; ABI/INFORM; JSTOR; ScienceDirect	Wed of science	EBSCO
Type	Peer-reviewed journals; recent Working papers	Peer-reviewed journals; Practitioner-Oriented Journals	Peer-reviewed journals	Peer-reviewed journals	Peer-reviewed journals; Harvard Business Review	Peer-reviewed journals; VHB Jourqual 3 rating of at least “B″	Peer-reviewed journals; ABS ranking
Sample	35	150	20	104	219	40	114

This study aims to fill this research gap by conducting a SLR of 272 peer-reviewed articles on BMI, exploring the phenomenon of BMI, and analyzing its antecedents, processes, types, barriers, outcomes, and moderators/mediators. This paper identifies the gaps and limitations of BMI in theory and practice, and proposes a clear and comprehensive theoretical framework to help practitioners of various types of enterprises make wise decisions and implement effective strategies for BMI in different environments and industries.

This study makes three contributions to the existing literature: First, unlike previous literature reviews that mainly focused on one or two aspects of BMI [11,12], our review focuses on revealing all aspects of the BMI phenomenon, such as the antecedents, processes, types, barriers, outcomes, and moderators/mediators of BMI, responding to the call of [8] and contributing to the theoretical accumulation and development in the field of BMI. Second, this paper constructs a theoretical framework of BMI that includes six main elements (antecedents, processes, types, barriers, outcomes, and potential moderators or mediators), and explains their relationships and mechanisms of action. This not only provides opportunities for academic researchers to explore BMI in depth, but also provides clear guidance for enterprises on how to conduct BMI. Third, based on a critical analysis of the existing literature, this study proposes important issues and directions for future BMI research, as well as some implications and suggestions for BMI practice.

This study proceeds as follows. Section 2 provides a theoretical background to BMI, while Section 3 explains the methodology used, including a detailed description of the SLR procedure employed. Sections 4, 5 present the results of an SLR and the theoretical framework, and Section 6 explores potential future research directions based on these findings and the theoretical framework. Lastly, Section 7 summarizes the conclusions of this study and its limitations.

## 2 Theoretical background

BMI refers to how a company creates and captures value by making novel, non-trivial changes to key elements or architecture of the BM [8]. Unlike traditional product, service, and technology innovations, BMI, which is considered as an innovation replacement or complementary [14], is tied to the way a company restructures its business and if it is successfully implemented, it can help companies gain a long-term competitive advantage [14,15]. In addition, BMI can help companies adapt to rapidly changing market conditions and help them survive and thrive in a volatile business environment [15]. Therefore, as an emerging field, BMI has attracted significant interest from scholars. Given the ever-changing customer needs and rapid technological advances [4], scholars have provided various insights from their respective fields on how companies modify their BMs and the outcomes that result from such modifications. However, there is still a limited understanding of BMI [8]. The reason for this is the lack of an appropriate framework and tools to support BMI efforts today [16]. There are now many SLRs of the BMI literature, such as [17], which summarizes the BMI literature in terms of antecedents, processes, and effects [8], which reviews and evaluates the BMI literature in terms of BMI concepts, processes, outcomes, and results, and [4] review 104 conceptual and empirical articles on BMI research from 2000 to 2017 in terms of drivers within the firm. [18], on the other hand, provide a systematic summary of the BMI process [19]. look at the micro-foundations in terms of human resource management to develop BMI by providing a comprehensive overview of the competencies required for BMI. However, none of these literature reviews have reviewed the antecedents, processes, barriers, types, outcomes, and potential moderators or mediators of BMI in an integrated manner. In conclusion, a new, comprehensive, systematic, and prospective review of the BMI field is currently lacking. For this reason, we propose an integrated theoretical framework to enhance the understanding of the relationship between BMI, its antecedents, processes, barriers, types, outcomes, and potential moderators or mediators.

## 3 Research design

**Fig. 1:**
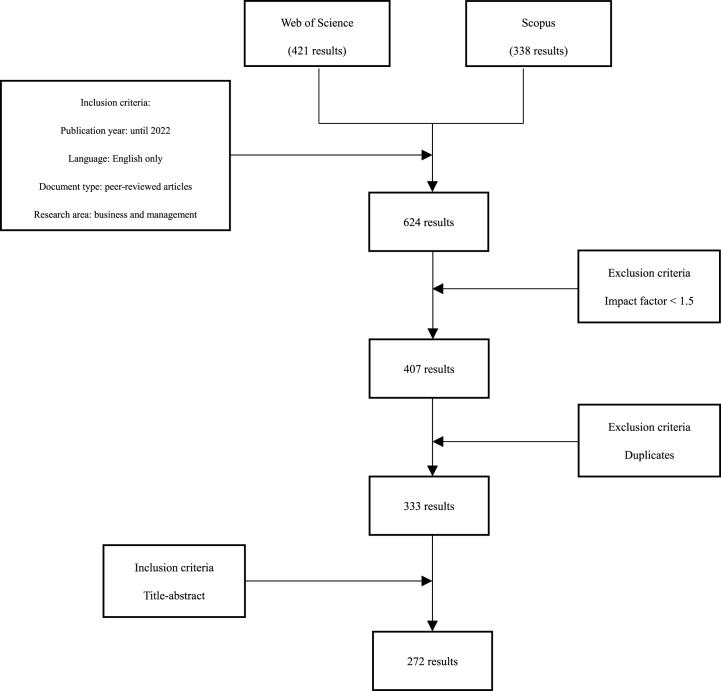
Literature search strategy.

The existing academic literature on BMI is scattered in various fields [18]; therefore, in this study, we chose to conduct an SLR. Because unlike traditional narrative literature reviews, SLR build on existing research, use clear and reproducible criteria, and are transparent in their process [[21], [22], [23]]. Moreover, it avoids some of the implicit biases of traditional narrative methods [24,25] and provides a stable basis for drawing reliable conclusions. Following the three stages of SLR proposed by Ref. [22], we first identified the research objectives, and then, designed the literature review process accordingly. Our main objective was to clarify the current state of research on BMI and to identify gaps and inconsistencies in this literature. Therefore, limiting the selection of papers to peer-reviewed journals is desirable [26]. We searched for relevant papers in the Web of Science and Scopus portals. These databases were chosen because they have high reliability in metadata and are widely used in the field of management [27,28]. The keyword used for the search was “BMI.” The search was limited to peer-reviewed papers in English [27], but without any search restrictions based on publication time to include all relevant literature. In the second stage, the papers were carefully selected to ensure that they focused on BMI as a research theme. Therefore, while searching the literature on BMI, the Boolean operator AND was used to check if the keyword “BMI” was included in the titles, abstracts, and keywords of the search results. Based on the above criteria, 624 samples were obtained. There are studies suggesting that only papers published in top peer-reviewed journals should be investigated [27], so we aimed to reduce the sample using the impact factor [28,29]. Following the proposal of [29], we targeted only the studies published in journals with an impact factor of 1.5 or higher. Through this criterion, the sample was reduced to 407. After the duplicates were removed, 333 samples remained. After conducting a thorough review of titles, abstracts, and keywords of various papers, a sample of 272 papers was selected for inclusion in the study (as shown in Fig. 1). The final step involved presenting the findings of the analysis. To provide a comprehensive theoretical foundation, the literature on BMI was reviewed, resulting in the division of the research stream into four distinct areas: antecedents, consequences, barriers, and processes of BMI. To provide a theoretical framework that can comprehensively clarify research trends, gaps, and inconsistencies in BMI literature, we developed a theoretical framework incorporating antecedents of BMI, moderating/moderating factors influencing antecedents and processes, processes, barriers, types, moderating/moderating factors influencing processes and outcomes, and outcomes based on the results of the review.

## 4 Descriptive analysis of a systematic literature review

This section presents a comprehensive overview of the evolution of BMI literature. To accomplish this, a range of metrics have been analyzed, including publication frequency, research methodology, journal distribution, geographic spread, and research trends [30]. This descriptive analysis seeks to evaluate the current state of the literature, as well as highlight potential areas for future research. We conducted a short bibliometric review to complement our SLR, providing a quantitative overview of the evolution and distribution of the BMI literature across time, across journals, across countries, and across research trends.

### 4.1 Frequency of publications over time

An analysis of the subject literature reveals that the first two papers on BMI were published in 2006. One of them examined how a family-owned firm specializing in nonferrous alloys changed its initial BM through technological innovation to become globally competitive in the face of a severe financial crisis [31]. The other, through a review of Napster and BitTorrent, showed that online piracy stimulates the development of BMI [32]. As can be seen in Table 1 below, only three papers were published in 2010, four years after the two papers were published in 2006 (through 2010). These three papers are primarily aimed at understanding the antecedents, barriers, and outcomes of BMI. For example, food companies threatened by high competitiveness due to economic recession and liberalization (Sosna et al., 2010) can learn through trial and error, continuously fine-tune their BM, and ultimately make much higher profits than their competitors, thereby creating successful, innovative BM that build and internationalize their enterprises. Even latecomers that are disadvantaged in terms of technological capabilities and market resources can successfully introduce disruptive technologies from developed countries to emerging countries through BMI. They are provided with cheaper, simpler, but adequate quality products and services that are readily available and accessible to the general public in emerging countries (Wu et al., 2010). Currently, the main obstacle to BMI is the conflict with existing assets and BM, and to address this conflict, Chesbrough (2010) suggests introducing an experimentation and effectiveness evaluation process within the organization. Although these three papers paved the way for the development of BMI, until 2016 there was still relatively little research on BMI, and since 2017 there has been a surge in the literature on BMI. This was prompted by the publication of a review of BMI by Foss & Saebi in 2017. This review details the concept, process, results, and outcomes of BMI, and identifies the research gaps that future researchers may address.

The analysis also reveals the existence of a theoretical period from 2006 to 2018, followed by an empirical period from 2019 to 2022. Although empirical papers on BMI appeared as early as 2012, theoretical studies still dominated until 2021 due to the early stages of BMI's theoretical development and the methodological challenges it faced. However, as interest in BMI has increased, the total number of quantitative studies has shown a rising trend, as seen in Table 1. Moreover, the number of empirical studies has increased by 80% over the five-year period from 2017 to 2022, indicating a shift from qualitative to empirical research in the field of BMI. In conclusion, this suggests that scholars are moving toward testing the proposed theories through empirical studies.

### 4.2 Analysis of research methods

Our review found that of the 272 articles selected, 169 (62.1%) used qualitative research methods, 98 (36.1%) used quantitative research methods, and 5 (1.8%) used a combination of qualitative and quantitative methods. This result indicates that qualitative research methods dominate the field of BMI, which may be related to the exploratory character of these studies, since BMI, as a relatively emerging topic, still lacks a mature and well-developed theoretical foundation and requires qualitative research to reveal its connotations, characteristics, and mechanisms, as mentioned in the previous section. Among the qualitative studies, the most commonly used method is case study, with 140 articles using this method, accounting for 82.8% of the qualitative studies, indicating that case study is one of the most effective and popular qualitative research methods in the field of BMI, which can provide rich and detailed insights through in-depth analysis of specific practice contexts. This is followed by conceptual studies (16), semi-structured interviews (9), Delphi scenario simulations (2), and action research (2), which can also explore issues related to BMI from different perspectives and dimensions. Among the quantitative studies, the most commonly used methods were various types of regression analysis (39 articles) and structural equation modeling (47 articles), accounting for 39.8% and 48% of the quantitative studies, respectively. Other methods, such as fuzzy ensemble qualitative comparative analysis (6 articles), Copula modeling (1 article), correlation analysis (1 article), cluster analysis (3 articles), and ANOVA (1 article), were also used in the reviewed articles, but relatively few of them may be related to the scope of application, complexity, and operability of these methods.

### 4.3 Journal distribution

We identified the journals in which a relatively large number of BMIs are published, and how well they are distributed across the journals (Table 2). The analysis revealed that a large number of articles were published in the *Journal of Sustainability* (31, or 11%). The other three journals with the largest number of published articles were the *Journal of Cleaner Production*, *Journal of Business Research*, and *Technovation*. These journals published 31, 29, and 24 papers, respectively, for a total of 84 (30%). The rest of the papers are scattered in various journals in different disciplines. This highlights the multidisciplinary interest in the field [33]. The remaining papers were scattered across various journals in different disciplines. The papers published in these three journals illustrate the recent trend toward introducing a sustainability perspective to BMI. This is evidenced by the *Journal of Cleaner Production*. [34], for example, propose BMI as a promising approach to improve the sustainability of manufacturing companies. They extend the existing research on BMI from the perspectives of value proposition, creation, acquisition, and delivery to a new perspective of unearned value, providing manufacturing stakeholders with the opportunity to identify the value that induces sustainable BMI.

**Table 2:**
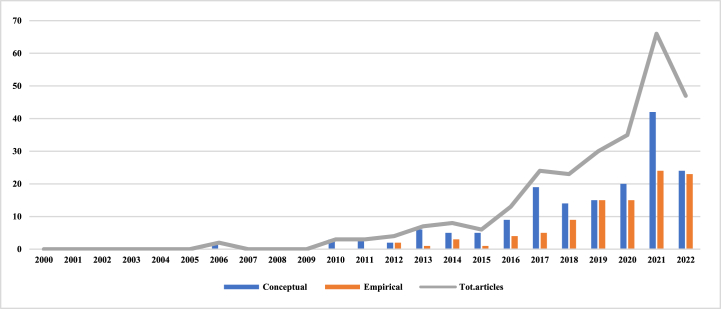
Article frequency by type of contribution and year.

### 4.4 Geographic spread

Table 3 demonstrates the participation of scholars from 41 countries in the discussion on BMI, indicating its global significance, both in developed countries such as Germany, the United States, the United Kingdom, and the Netherlands, as well as in emerging countries such as China and Indonesia. The highest number of scholars came from China, but most of the research in this area has been conducted in developed countries, particularly in the United States, the United Kingdom, Sweden, Italy, and Germany. This is likely due to the advanced Internet technologies and government initiatives, such as the German government's strong support for Industry 4.0 and the Swedish government's emphasis on sustainable development. Conversely, research in this field has been largely neglected in emerging and in Far Eastern countries, with China being the exception. Additional research needs to be conducted in these countries to account for possible publications in non-English languages and in journals not indexed by Scopus. Replicating studies in various contexts is crucial to ensure the generalization and validity of the research results [35]. This would address the problem of generalization caused by single geographies [36]. Thus, we strongly encourage researchers from underrepresented or non-represented countries to expand their work in this field, which would provide valuable new insights and deepen our understanding of BMI in unexplored contexts (see Table 4).

**Table 3:**
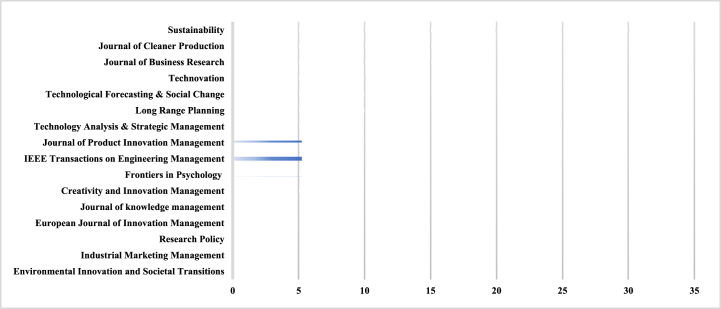
Journals of the selected articles.

**Table 4:**
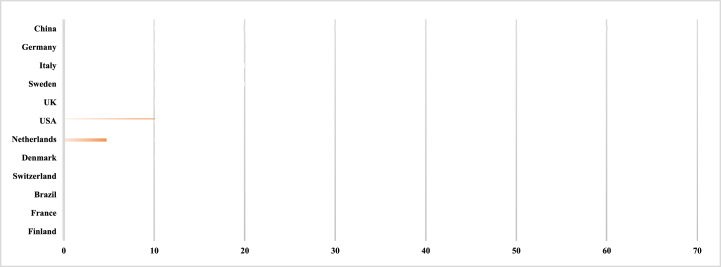
Countries' production on the basis of authors’ affiliations.

### 4.5 Research trends

**Fig. 2:**
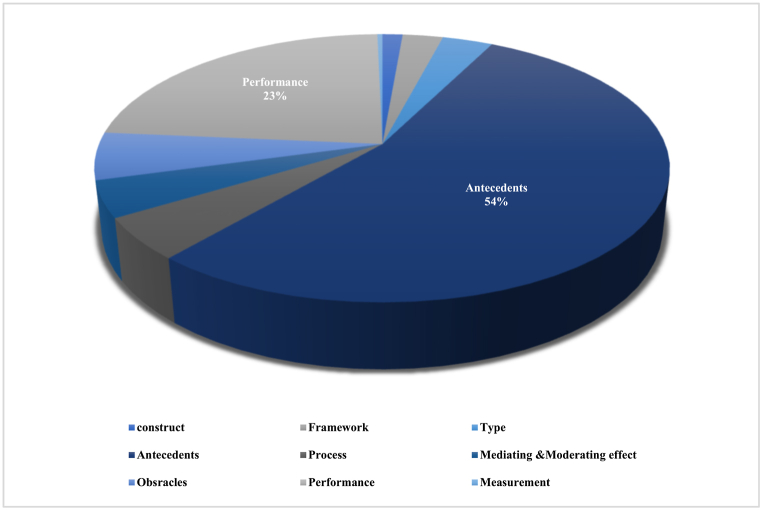
Research trends.

As shown in Fig. 2, most research on BMI focuses on its drivers and antecedents, accounting for 54% of the studies. The outcomes of BMI follow closely with 23% of the research. However, there is limited research on its barriers, processes, mediating effects, and types. This shortage of research on these aspects may be because the current BMI research is still in its early stages and lacks proper theoretical foundations [8]. Thus, future research should concentrate on the processes and mediating effects of BMI to advance its practical implementation.

## 5 Content analysis of a systematic literature review

In this section, we present the results of a content analysis of SLR. Due to the ambiguity in the current literature and the dynamic nature of BMI, this study has developed a theoretical framework of antecedent-process-outcome to clarify the research trends in BMI and identify gaps, inconsistencies, and future research directions. Along with examining the antecedents, processes, and outcomes that may influence BMI, this theoretical framework also considers the barriers, types, and moderating/mediating variables that can affect the BMI process and outcomes. To the best of our knowledge, this is a point that has not been considered in previous studies. Furthermore, our theoretical framework has led us to develop a research agenda that offers practical suggestions for addressing gaps and limitations in the literature.

### 5.1 Antecedents of business model innovation

#### 5.1.1 Technology-driven perspective

Research on the antecedents of BMI has been very active in the last 20 to 30 years, but systematic studies are lacking. Although it is believed that BMI is influenced by technological advances and environmental changes, the antecedent studies do not link these changes to BMI. In fact, BMI is a result of the rapid development of information technology. In order for companies to get users to accept technology that has no objective value, they must choose the appropriate BMI to bring the technology to the market [37]. Therefore, some researchers have started to focus on the impact of technology on BMI. For example, a qualitative study conducted by Ref. [38] on 68 SMEs in Germany suggests that Industry 4.0 may have an impact on the BMI of SMEs in manufacturing. Meanwhile [39], find that the emergence of AI is associated with BMI through a detailed case study of six major industrial companies engaged in digital services. In addition [40], found a direct positive impact of big data analytic capabilities on BMI using survey data from 253 companies in the UK and through qualitative comparative analysis of PLS-SEM and Fuzzy Set Qualitative Comparative Analysis.

Although there is a wealth of research based on technology-driven perspectives, many studies overemphasize BMI as a means of technology commercialization, ignoring the fact that BMI is actually a strategic change at the firm level. Furthermore, technology development requires significant investment, which may lead firms to focus too much on technology itself and neglect BMI, making them stubborn in their BM and difficult to adapt to market changes. Therefore, companies should find a balance between technology and BMI to achieve long-term business success. However, existing studies have not conducted in-depth research on the balance and correlation between technological innovation and BMI. In addition, the need for technology is not consistent across companies, and existing research does not clearly analyze the extent to which different technologies affect different companies, making it difficult for entrepreneurs and managers to judge the applicability of new technologies.

#### 5.1.2 Strategic-Driven Perspective

There are some differences between technology-driven perspectives that focus on the instrumental attributes of BMI and researchers with a strategic perspective. Researchers with a strategic perspective emphasize that BMI should be adapted to the environment in which the company operates. BMI has been examined in various contexts in the existing literature. [41] investigated the incidental factors underlying the acceptance of BMI at the bottom of the pyramid in India, while [42] analyzed the reasons for BMI in Nigeria’s financial services industry. [43] analyze the key factors of BMI in frontier markets. [44] research confirms that the co-working spaces of a company have a high likelihood of stimulating BMI. Furthermore, the operation of a company is more or less influenced by politics. Extant studies have pointed out that factors such as regulatory uncertainty [45], regulatory systems [46], and policy combinations can promote a company's BMI [47]. As the global population grows and food and energy supplies become increasingly scarce, more and more studies are focusing on circular BMI to alleviate the pressure caused by population growth and protect the environment. Circular economy BMI places a greater emphasis on sustainable resource use and waste reduction than traditional BMI, with the aim of minimizing the environmental impact of economic activities [48]. Although many companies have a strong interest in circular economy BMI, few established companies have actually successfully implemented it [6]. Circular economy BM is a complex innovation challenge [6,49] requiring companies to change their key operations in uncharted territory and to contend with dominant business paradigms [6]. To advance the practice of circular BM [48], embed sustainability, circularity, and BMI in the framework of design thinking. Studies have shown that companies that prioritize sustainability are more likely to adopt BMI, as observed in the agricultural sectors of Sweden [50] and Finland [51].

Another core logic of the strategic perspective is based on a company's existing resources and abilities, and it advocates for the company to take advantage of its strengths, avoid its weaknesses, and only do what it is good at and what is within its capabilities. However, with the company's existing resources and abilities, BMI is difficult. To break the limits defined by the existing resources and abilities for BMI, more companies are developing DCs(dynamic capabilities) and hope to overcome organizational inertia via it. The empirical studies by Refs. [52,53] have also confirmed that DCs can facilitate BMI more reliable. Furthermore, as global value chains become more complex and decentralized, sources of knowledge for BMI are becoming increasingly fragmented. This includes knowledge from global sources [54], as well as customer knowledge [55] and other sources. Therefore, it is crucial for companies to develop external knowledge management capabilities [56] and conduct external knowledge searches [57] prior to engaging in BMI, in order to access the fragmented knowledge sources necessary for successful innovation. While the strategy-driven perspective provides a detailed discussion of how firms analyze their internal and external environments to access BMI opportunities, research from this perspective, as well as the technology-driven perspective, has certain limitations. These include an excessive focus on environmental impacts, inadequate attention to the internal elements of the firm, a lack of research on the essential mechanisms of value creation, limited exploration of alternative strategies for BMI, and a neglect of consumers and customers as a source of innovation.

#### 5.1.3 Demand-driven perspective

The starting point for BMI is the creation of value through activities that cater to the needs of customers. However, researchers with a strategic outlook have focused excessively on a company's intentions, resources, capabilities, and past successes, neglecting to address customer needs [58]. As a result, some researchers have shifted their attention to the needs of target or potential customers. The literature presently concentrates on the needs of target customers [59], unfulfilled needs [60], and significant characteristics of customers that stimulate BMI [61]. This shift has been gradual, moving from high-end developed countries to low-end developing countries since consumer demand in developing and underdeveloped nations (bottom of the pyramid) is still unmet and represents a potential market of great interest to both existing and emerging companies.

Additionally, studies have explored the role of stakeholder preferences and behaviors, both within and outside the industry, in driving BMI [62,63]. Since stakeholders within and outside the industry are also potential customers of the company, examining their preferences and behaviors can provide insights into the company's BMI. Positioning perspective studies on the facilitators of BMI are also a subject of discussion. Scholars contend that BMI is driven by both active and passive market orientation [64,65] Despite the demand-driven perspective emphasizing the importance of firms in uncovering demand, it is still unclear how firms identify customer needs. While this approach is more straightforward and easier to comprehend, there is still a gap in research in this area. Thus, more extensive research is required to analyze and understand how firms identify customer needs.

#### 5.1.4 System-driven perspective

According to some scholars, BMI is a complex process that cannot be fully explained by a single perspective. As a result, a mixed perspective has been proposed to systematically explain the impact of multiple factors on BMI. For instance Ref. [66], analyzed semi-structured interviews with 22 Finnish firms and discovered that firm participation in BMI is influenced by several factors, including the decline of leading firms, regulation, and competition. These factors work together to affect firms' decisions and actions regarding BMI. Similarly [67], examined the BMs of six organizations offering healthcare services at the bottom of the pyramid (Bop) and identified several factors that facilitate inclusive healthcare delivery, such as co-creation of patient demand, community engagement, and strategic partnerships. These factors interact with each other to form the BM that enable inclusive healthcare delivery. Although a mixed perspective can explain the impact of multiple factors on BMI, further research is necessary to understand the interrelationships and coordination among different factors to gain insight into the underlying drivers of BMI.

#### 5.1.5 Top management-driven perspective

In order to gain a more thorough comprehension of why companies choose to participate in BMI, they must analyze the factors that lead to BMI from an intra-organizational viewpoint. On one hand, it is imperative to investigate how top management's cognition, abilities, and actions affect BMI from the company's standpoint. Previous studies have shown that top managers' cognitive ability [68,69], narrative ability [70], management skills [71], management bonds [72], DC [15], leadership style [73], personal connection, various behaviors across industries [74,75], and diversity in the top management team [76] are among the factors that contribute to BMI. However, these studies have focused solely on the competencies and actions of top corporate leaders and have neglected to consider the impact of employees on BMI in terms of their human capital, skills, and psychographics (such as motivation and engagement). Employee competencies and actions play an equally crucial role in the success of BMI, so researchers should expand their research to include employees. On the other hand, researchers should also investigate the role of organizational design in BMI from an organizational design perspective. Although [8] have noted that organizational design is critical to the success of BMI, the current review has not identified any studies that have examined the antecedents of BMI from an organizational design perspective.

Despite extensive research on the antecedents of BMI in previous studies, researchers have tended to overestimate the impact of external factors on BMI and neglect the fact that BMI is ultimately intended to be profitable. They have also failed to investigate the underlying motivations for BMI. As a result, we believe that researchers should revisit previous research findings and take a broader and more systematic approach to exploring the antecedents of BMI. Additionally, discrepancies in the identification of BMI antecedents among researchers suggest the need for careful comparison and analysis of various research methods and results in future studies. Ultimately, research on the antecedents of BMI should incorporate multiple factors from both within and outside the firm in order to provide a more comprehensive understanding of the motivations and drivers behind firm participation in BMI.

### 5.2 Moderating and mediating variables affecting the antecedents of business model innovation

Extensive research has explored the contributing factors to BMI, but its relationship with BMI is influenced by several moderating and mediating factors. Environmental factors are important moderators that shape the effectiveness of BMI [[77], [78], [79], [80]], alongside other factors such as entrepreneurial recognition, market information integration ability, and strategic sensitivity. However, the literature tends to neglect the effects of institutional and industry context on BMI effectiveness when examining these moderating factors. Thus, future research should account for these factors.

Furthermore, research has shown that some of the preconditions of BMI have indirect effects on BMI through mediating variables such as reconfiguration ability [80], opportunity recognition ability [44], learning ability [81], and resource integration ability [82]. However, current research has neglected the impact of organizational structure and human capital on these mediating roles. Therefore, future research should explore these under-explored areas to gain a more comprehensive understanding of the complex relationships between the antecedents and effectiveness of BMI. Such research can inform theoretical development and management practices that promote innovation within firms.

### 5.3 Process of business model innovation

#### 5.3.1 Component approach

Researchers studying the BMI process need to determine whether to develop a new model from scratch or modify an existing one, taking into account various perspectives and contexts. In the past, early studies focused on changes in BMI components, but with the emergence of digital technology, scholars such as [83] have integrated digital technology dimensions into the nine elements of the BM canvas to reflect changes in BMI. Furthermore, the platform economy has made changes in components more complex, such as value propositions, products, partnerships, and profitability models [84], while trends in sustainability have also influenced changes in BMI components [85]. While focusing on component variations aids in BMI process study, the lack of agreement among scholars on BMI components has impeded BMI development. Future research must take a more systematic approach to explore all facets of the BMI process, including interactions between components and the relationship between components and the overall BMI process.

#### 5.3.2 System approach

Many researchers suggest that a systems approach perspective is necessary to gain a more comprehensive understanding and assessment of the effectiveness of BMI implementation, due to the multiple dimensions and complexity of a company's BMI process. This perspective views BMI as a dynamic, complex system comprised of various interdependent elements, such as the environment, organizational structure, resources, human capital, technology, and markets, which collectively influence the success of the BMI process through continuous adjustment, evolution, and change. In this view, the BMI process is regarded as a system of activities, with different activities added, modified, and discarded over time [86]. proposed this perspective, arguing that companies need to continually adapt and adjust their strategies and operations through trial and error to achieve the BMI goals.

Alternatively [87], visualized the BMI process from a system dynamics perspective, showing that it is a complex and dynamic system in which different factors interact and influence the effectiveness of BMI implementation. Their study highlights the importance of the systems approach in BMI research and provides a novel method for understanding and analyzing the BMI process. However, the literature on the BMI process from a systems perspective is limited, and further research is necessary to expand our understanding of the BMI process. Additionally, it is important to note that while the systems approach provides a comprehensive perspective, it may not be applicable to all situations, and the choice of approach must be made on a case-by-case basis in practical applications.

#### 5.3.3 Learning and experimental approach

Many scholars argue that BMI is an ongoing process of individual and organizational learning, experimentation, and trial and error, similar to other types of innovation [[88], [89], [90], [91]]. This process aims to identify and develop new business opportunities while gradually adapting and refining the BM. From this perspective, the process of BMI is constantly evolving and requires continuous reflection and adjustment to ensure that the BM remains aligned with market needs and creates value. However, few studies have evaluated the effectiveness of learning and experimentation processes in the BMI process [8]. and others suggest that past evaluations of the effectiveness of learning and experimentation in the innovation process have tended to rely solely on economic outcomes as evaluation criteria, which is not sufficient. Therefore, scholars need to develop more complete and systematic evaluation criteria that link the goals and processes of learning and experimentation to the outcomes and impacts of BMI to assess its effectiveness in an integrated manner.

Moreover, many studies on learning, experimentation, and trial-and-error processes only describe the steps and processes of learning and experimentation in BMI without exploring the sources of learning and experimentation programs or how to control the learning and experimentation processes. Therefore, there is a need to further explore the effectiveness of learning and experimentation in BMI, including the sources, methods, and processes of learning and experimentation, and the ways to control the learning and experimentation process. This will provide a more comprehensive and systematic theoretical basis for the BMI process, enabling it to adapt to changing market demands and maintain a competitive edge.

#### 5.3.4 Steps approach

While some scholars suggest that the BMI process should involve learning, experimentation, and trial-and-error, others have outlined specific steps and phases. For example [92], propose four phases of the BMI process: initiation, conceptualization, integration, and evaluation. Similarly [93], identify four key steps in the BMI process, including assessing the environment for new opportunities, conveying a sense of urgency, experimenting with new opportunities, and using intuition and data to make decisions [94]. describe the evolution of Sony PlayStation's BMI in four phases: establishing an installed base, building an online network, leveraging the network, and exploring new models. In addition, some scholars have studied the role of key competencies such as DC, responsiveness, reversal, prediction, and operationalization [68] in the BMI process. However, researchers have not yet examined how competencies and resources differ at different stages of the BMI process, or how to address threats that arise during the different processes. This theoretical gap needs to be addressed in future research. Additionally, successful BMI cases are scarce due to confidentiality concerns, making it critical for future research to focus on this area and explore strategies for achieving better BMI. We suggest that companies should develop their own BMI strategies based on their unique circumstances, including their environment, risks, resources, and leadership styles, to respond to market changes and gain a competitive edge. It's worth noting that different situations may require different BMI strategies and processes, and as such, various theories and methods should be applied flexibly throughout the BMI process.

#### 5.3.5 Types of business model innovation

When it comes to BMI, it's crucial to consider not only the creation of a new BM or the modification of an existing one but also the type of BM, since the classification of BMI forms the basis of BMI theory development [8]. Various classifications for BMI have been proposed by scholars, with some based on the novelty of the BM such as evolutionary, focused, adaptive, and complex BMI [95], while others are based on industry and business practice phenomena. For example, platform skimming, platform revenue generation, and platform coordination are three platform-based BMI influenced by the Internet economy [96], while sharing-based BMI is based on the impact of the sharing economy [97]. Meanwhile, technology-based, value network-based, and financial barrier-based BMI are proposed based on Kodak and Microsoft's failure in the gaming market [98], and imitative “good enough” BMs, RenQing BMs, MianZi BMs, and hybrid BMs are constructed based on Chinese consumers' unique consumption behavior [99]. Furthermore, social BMI is proposed by Ref. [100] to improve human welfare and fulfill social missions, while [101] use five actual zero-carbon building projects to develop a typology of BMI in three theoretical dimensions: value provision and related project processes, stakeholder networks, and institutional boundaries.

Although classification criteria based on actual phenomena have contributed to the theoretical construction of BMI, their weak theoretical foundation, vague classification criteria and unclear boundaries make it difficult for researchers to provide better insights about BMI for different companies. Although it is not clear what type of BMI should be used by different types of companies in the 272 papers we reviewed, we summarized a table of research on BMI for established companies, SMEs, family-owned companies, start-ups, and multinational companies in order to help operators of different types of companies achieve better BMI (see Table 5). From Table 5, we infer that different types of organizations can adopt different types of BMI depending on their context and industry. e. For example, established firms are more likely to pursue more radical or disruptive BMI through value network innovation, extensive external search, challenging or changing existing industry structures or value propositions, such as Netflix's streaming service model, Apple's iTunes, the App store, and Amazon's Prime membership and AWS services [102]. Social purpose organizations may prefer eco-innovation or social innovation in emerging economies to address social and environmental issues and achieve the dual mission of creating social and economic value [53]. For example, circular BMI, green BMI, and so on. Multinational enterprises may prefer to engage in global or localized BMI to adapt to market demands and cultural differences in different countries and regions, as illustrated, for example, by the localization of the supply chain of Zongteng, a Chinese cross-border e-commerce company. SMEs may pursue more incremental or adaptive types of BMI to exploit their flexibility or niche markets. Family firms, on the other hand, may pursue a more conservative or traditional BMI to preserve their core values or family heritage as illustrated by Ref. [31] case study of a family firm specializing in non-ferrous alloys and [103] recommendation to use the family's unique culture and relationship network to maintain brand image and reputation. However, these types of BMI are not mutually exclusive, and some organizations may combine or switch between different types of BMI depending on their strategic goals, market opportunities, or environmental changes. For example [104], case study of carpet manufacturer Interface shows how the company adopted different types of BMI to achieve sustainability goals, such as product service system innovation, technological innovation, social innovation, and organizational innovation. While the above provides some useful information and references, there may be biases or errors. After all, these methods do not consider the influence of cultural, political, and economic factors on BMI in different countries or regions, among others. Therefore, there is a need for researchers to develop a comprehensive and systematic taxonomy to better understand and examine the factors involved in selecting and implementing different types of BMI in different types of organizations from different backgrounds and industries and what types of companies are best matched with what types of BMI.

**Table 5:** A summary of BMI research on different company types.

Author	Research Area	Company Type	Research Methods	Conclusion
[61]	Antecedents	Established firms	Conceptual	Potential consumers with a mix of characteristics associated with their own creativity
[57]	Antecedents	Established firms	Quantitative: Gaussian copula model	Extensive external search
[97]	Types	Established firms	Single case study	The content and model of shared BMI
[98]	Types	Established firms	Multiple case studies	Technology, value networks and financial hurdle rates
[41]	Antecedents	Social Purpose Organization	Single case study	Stakeholder Stability and Stakeholder Motivation
[53]	Antecedents	Social Purpose Organization	Multiple case studies	Dynamic capabilities: sense, seize and transform
[105]	Antecedents	Social Purpose Organization	Multiple case studies	Persuasive techniques
[106]	Antecedents	Social Purpose Organization	Multiple case studies	Digital
[86]	Process	Social Purpose Organization	Multiple case studies	Activity System Perspective
[107]	Process	Social Purpose Organization	Single case study	Origins and early growth, the search for social entrepreneurship, and the shift to a new model
[108]	Process	Social Purpose Organization	Single case study	Creation period, growth period, challenge period and trek period
[109]	Antecedents & Outcomes	Social Purpose Organization	Multiple case studies	Various Stakeholders & Overcoming tensions and achieving social value and financial stability
[110]	Outcomes	Social Purpose Organization	Quantitative	Social and economic performance and organizational legitimacy
[111]	Antecedents	SMEs	Quantitative: SEM	Digital Platform
[112]	Antecedents	SMEs	Quantitative	Learning ability, consumer demand, entrepreneurship and website performance
[113]	Antecedents	SMEs	Quantitative: PLS-SEM & fsQCA	Customer relations, supplier relations, competitor relations and knowledge relations
[76]	Antecedents	SMEs	Quantitative	Functional diversity and tenure diversity in TMT
[74]	Antecedents	SMEs	Quantitative: fsQCA	TMT transgressive behavior
[114]	Antecedents	SMEs	Quantitative: PLS-SEM & fsQCA	Open innovation and organizational agility
[115]	Antecedents	SMEs	Quantitative: SEM	Organizational complexity plays a mediating role between Enterprise Resource Planning and BMI
[73]	Antecedents	SMEs	Multiple case studies	Directive Leadership, Empowering Leadership
[52]	Antecedents	SMEs	Quantitative	Different dynamic capabilities influence different aspects of BMI in German Mittelstand SMEs
[116]	Antecedents	SMEs	Quantitative	Organizational Values: Flexibility, rational
[70]	Antecedents	SMEs	Single case study	Slow Storytelling
[56]	Antecedents	SMEs	Quantitative: PLS-SEM & fsQCA	External Knowledge Management Capability
[117]	Antecedents	SMEs	Multiple case studies	Open Innovation
[38]	Antecedents	SMEs	Multiple case studies	Industry 4.0
[118]	Antecedents	SMEs	Quantitative	Social capital, organizational learning capabilities, entrepreneurial orientation
[119]	Antecedents	SMEs	Multiple case studies	Effective control logic
[120]	Antecedents	SMEs	Single case study	Long-term perspective, business strategy, influential enthusiasts, collaboration and so on
[121]	Antecedents	SMEs	Single case study	Dynamic business modelling approach
[68]	Antecedents	SMEs	Single case study	Four sensing abilities: react, flip, predict and plan
[122]	Antecedents	SMEs	Single case study	Technology Innovation
[123]	Antecedents	SMEs	Quantitative: PLS-SEM	Absorption capacity
[124]	Antecedents	SMEs	Single case study	Eco-Innovation
[93]	Process	SMEs	Multiple case studies	Four key BMI process activities: Assess, Communicate, Explore, and Process
[91]	Process	SMEs	Multiple case studies	Three main stages of BMI process: start, experimentation and replication
[125]	Obstacles	SMEs	Single case study	High cost of fixed assets, government regulations, weather and traditional
[126]	Obstacles	SMEs	Multiple case studies	Build legitimacy for their innovations, engage in joint value creation activities, obtain sufficient financial resources, and adapt to regulatory uncertainty
[127]	Antecedents & Outcomes	SMEs	Quantitative: PLS-SEM	Antecedents: Innovativeness and business environment Outcomes: Performance
[128]	Antecedents & Outcomes	SMEs	Semi-structured interviews & Quantitative	Antecedents: Entrepreneurial Value Intentions Outcomes: Financial and Social Performance
[129]	Antecedents & Outcomes	SMEs	Quantitative: PLS-SEM	Antecedents: Entrepreneurial Orientation Outcomes: New Product Development Performance
[130]	Antecedents & Outcomes	SMEs	Quantitative: SEM	Antecedents: Open Innovation Outcomes: Performance
[131]	Antecedents & Outcomes	SMEs	Quantitative: SEM	Antecedents: Internal factors (innovation activities and strategic orientation) or external factors (market and technological turbulence) Outcomes: Lead to changes in the strategy and structure of the company, improving the performance of SMEs and their ability to innovate
[132]	Process & Outcomes	SMEs	Single case study	The three processes of implementing BMI: leveraging, transforming and mobilizing resources. Outcomes: Responding to COVID-19 Crisis
[133]	Obstacles & Outcomes	SMEs	Quantitative: SEM	Outcomes: BMI does not directly improve firm performance, but it works through three mediating factors: efficiency growth, revenue growth, and organizational capability Obstacles: Limited resources, expertise and flexibility
[38]	Outcomes	SMEs	Quantitative	Create value
[134]	Outcomes	SMEs	Quantitative: PLS-SEM	Sustainable performance.
[135]	Outcomes	SMEs	Quantitative: SEM	Promoting the growth of manufacturing SMEs
[31]	Antecedents	Family Business	Single case study	Innovation Network
[89]	Antecedents	Family Business	Single case study	Trial-and-Error Learning
[103]	Antecedents	Family Business	Single case study	learning, market orientation, competency enhancement, & motivation & opportunity enhancement HRM practices
[136]	Antecedents	Family Business	Quantitative	Collaboration for Open Innovation and intergenerational clusters
[137]	Antecedents	Start-ups	Quantitative: fsQCA	Technology Mastery and Business Complexity
[35]	Antecedents	Start-ups	Multiple case studies	Lean Startup Method
[44]	Antecedents	Start-ups	Quantitative	Spatial creativity in co-working spaces
[138]	Antecedents	Start-ups	Multiple case studies	Exploratory activities, organizational flexibility
[80]	Antecedents	Start-ups	Quantitative: SEM	Entrepreneur Network
[79]	Antecedents	Start-ups	Quantitative: SEM	Executive cognitive ability, entrepreneurial bricolage
[139]	Antecedents	Start-ups	Quantitative: fsQCA	Different configurations of psychological and social capital
[140]	Antecedents	Start-ups	Quantitative	Entrepreneurial cognition: configuration cognition, willingness cognition and ability cognition
[141]	Antecedents	Start-ups	Quantitative: SEM	Work experience, founder's creativity
[142]	Antecedents	Start-ups	Quantitative	Space Creativity
[143]	Antecedents	Start-ups	Multiple case studies	ICT network characteristics, adapting to the local environment, balancing formal and informal governance mechanisms, and creating and connecting an ecosystem of partners
[144]	Antecedents & Outcomes	Start-ups	Quantitative	Antecedents: Value Proposition Innovation, consumer needs and preferences Outcomes: Performance
[145]	Outcomes	Start-ups	Quantitative	Business Survival
[146]	Outcomes	Start-ups	Single case study	Sustainable BMI
[147]	Outcomes	Start-ups	Quantitative	Helping to classify sharing economy companies
[148]	Outcomes	Start-ups	Single case study	Contributes to environmental and social sustainability
[149]	Outcomes	Start-ups	Quantitative	Inverted U-shaped relationship between BMI and sustainable development performance
[150]	Antecedents	Multinational Companies	Quantitative	R&D intensity, absorption capacity
[151]	Antecedents	SMEs & Multinational Companies	Multiple case studies	Strategic intent, culture, transparency of knowledge, acceptance of knowledge, complementary and conflicting assets
[152]	Antecedents	Multinational Companies	Single case study	Supply Chain Localization
[6]	Antecedents & Obstacles	Multinational Companies	Multiple case studies	The barriers and drivers of SBMI are identified at three levels of organizational design: institutional, strategic, and operational

#### 5.3.6 Obstacles of business model innovation

The process of implementing BMI is not always smooth. During this process, companies face various cognitive, resource, and capability constraints that can create serious barriers to effective BMI implementation. A new BM does not arise from an industry or company's existing model but is spun off from it. While existing models can inspire new BMs, they also limit management's perception of BMI to some extent. Management itself can be a constraint to BMI implementation, as narrow industry-centric perceptions and high information load can cause management to overly focus on direct competitors and incremental gains [153]. This may result in ignoring inter-industry differences and making poor decisions [154]. note that attempts to apply the successful pay-as-you-go model of the telecommunications industry to sustainable transportation companies failed due to not recognizing important marketing differences between industries and not taking into account the need to support the infrastructure required to support the new model. On the other hand [155], shows that the implementation of a new BMI failed in German utilities due to management's misunderstanding of solar energy [156]. also demonstrate that perceived differences between members of the innovation department and the core business within the company may inhibit the implementation of a new BMI. This is because companies have already invested significant resources and capabilities in their existing BMs and have a full and clear understanding of how the resources and capabilities of the existing model are linked together through rich practice. Furthermore, developing a new BM brings significant uncertainty and risk, so firms are hesitant to invest existing resources and capabilities in a new BM to maximize profits [157]. find that existing resources and capabilities largely constrain BMI, as illustrated by the case of the four largest European banks [158]. examine the costs, capabilities, and risks that limit the development of aggregated and centralized P2P (BMI) in the German utility sector. Although the researchers elaborate on the possible barriers to BMI implementation and their associated theories in terms of perception, capacity, and resources, they neglect socio-cultural factors such as customer awareness and behavior, political, legislative, and economic challenges of stability and ambiguity. Furthermore, these studies do not specify the mechanisms by which these barriers specifically constrain BMI implementation, nor do they suggest ways to overcome them.

### 5.4 Mediating variables affecting the outcomes of business model innovations

There is still controversy regarding the mediating variables that influence BMI outcomes. While past research has emphasized the positive impact of BMI on firm performance, not all types of BMI create a competitive advantage for firms and improve their performance. A recent study conducted a survey of 563 European SMEs and found that the direct association between BMI and firm performance was not significant. Instead, superior performance was achieved through the mediation of efficiency, organizational capacity, and revenue growth [133]. Another study conducted in 91 seafood processing SMEs in Thailand found that sustained competitive advantage mediated the relationship between BMI and sustained performance [134]. Although existing empirical studies reject the idea that BMI necessarily leads to superior firm performance, there is still relatively little research on the role of different mediators in the relationship between BMI and firm performance. Thus, further exploration is needed. Future studies should adopt a multi-country and multi-firm research design to ensure the accuracy and applicability of the findings. Overall, the relationship between BMI and firm performance is a complex issue that requires more in-depth research.

### 5.5 Outcomes of business model innovation

BMI is an essential tool for business innovation and has become an indispensable factor in achieving organizational success. This innovation can produce a range of outcomes, such as process and product innovation, sustainability, and improved economic performance. Current research shows that the outputs of BMI are not limited to the internal organization but also include the broader social and environmental domains. The role of BMI is even more pronounced in situations where firms are resource-poor. BMI can be used as a complement or alternative to organizational innovation [159], allowing firms to gain an advantage in a competitive marketplace. In addition, BMI can facilitate the development of areas such as process innovation [38], open innovation [160], and sustainability-oriented service innovation [161]. These innovations can not only contribute to economic growth [127], protect organizations from threats of environmental change [162], and reduce uncertainty [6] but also promote women's empowerment in poor communities [163], secure water [164], improve healthcare [67] and introduce a global perspective of sustainability into corporate business strategies [155,[165], [166], [167]]. However, from the available literature, few studies have answered why BMI leads to such results. Some scholars argue that it is because BMI is an inimitable resource that provides a competitive advantage to firms. However, this view is not sufficient. More in-depth research is needed to explore the mechanisms of BMI's impact on organizational success.

**Fig. 3:**
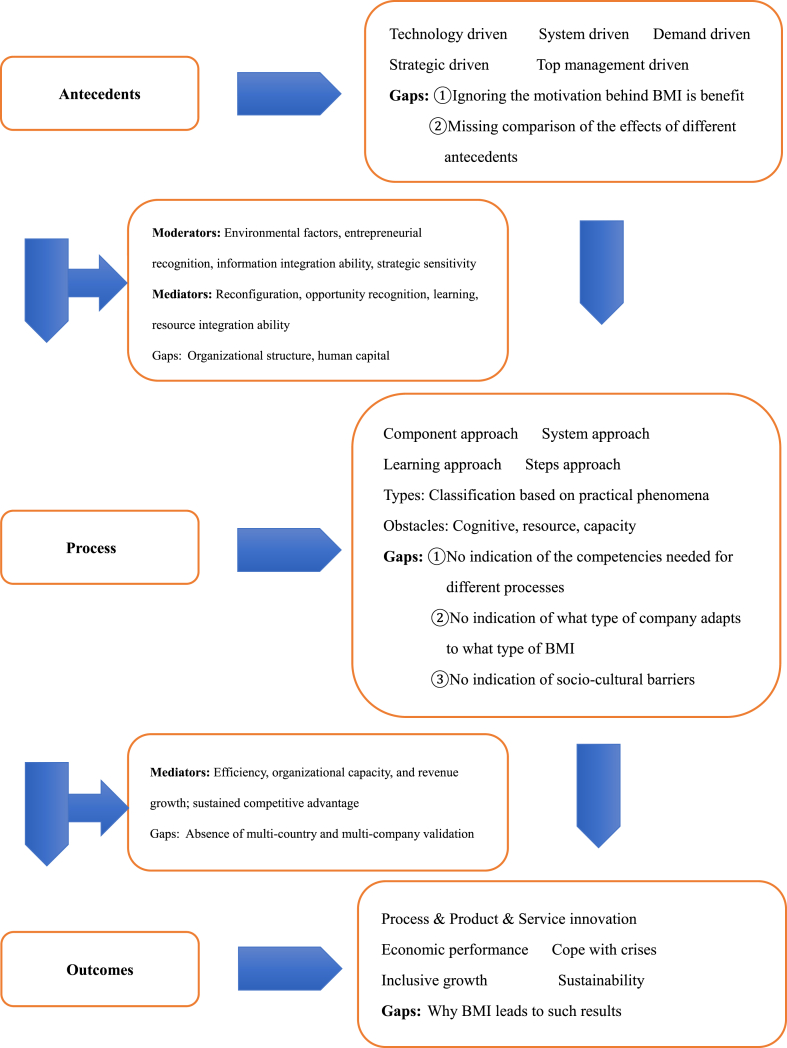
Antecedent-process-outcome theory model of business model innovation.

Overall, BMI is an important tool for corporate innovation that can contribute to the sustainability of the organization's internal and social environment. Although the existing literature recognizes the multidimensional value-creating attributes of BMI, more research is needed to reveal its impact mechanisms to promote the success of companies in achieving their sustainability goals. Based on the results of a content analysis of a SLR, we propose a theoretical framework of antecedent-process-outcome. Fig. 3 shows the framework of BMI. This theoretical framework can help organizations better understand the value creation mechanism of BMI and develop appropriate BMI strategies according to their own situations. Additionally, this framework can also help future researchers explore the characteristics of BMI and the value creation process more deeply.

## 6 Implications for future research

This paper presents a comprehensive review of the literature on BMI and highlights research gaps based on the available literature and a proposed theoretical framework. BMI is a relatively new field, and this study summarizes the antecedents, processes, barriers, types, outcomes, and potential moderating/mediating factors in BMI. The paper concludes with suggestions for future research to address the gaps in the current literature.

Considering the results of the SLR, the framework begins by summarizing several important drivers of BMI, including the redefinition of new technologies [38,39], the redefinition of established institutions and structures in the industry [46,47], and the presentation of new value propositions for different customers [[59], [60], [61]]. While these factors explore various aspects that may drive BMI in depth, they only provide an overview of BMI antecedents, and few studies take a dynamic perspective to dissect the journey of corporate BMI. Therefore, this paper encourages scholars to analyze how BMs of different firms in different contexts have changed over time to better understand how firms include technology, customer needs, top-level capabilities, and corporate resources in BMI. Moreover, existing research still has a limited understanding of how firms identify and propose new value propositions for BMI or other antecedents that influence BMI, especially across different types of firms. In the future, attention could be focused on the specific mechanisms by which different types of firms identify the driving elements of BMI. In addition, our review identified individual factors that may positively or negatively influence the relationship between BMI antecedents and their processes. Still, there is a lack of in-depth research on moderating or mediating effects that may affect BMI antecedents. Current research in this area has focused on environmental dynamics [79,102], with limited attention to the moderating role of institutional and political factors. Therefore, we argue that further research is needed to understand how these aspects can potentially moderate the impact of antecedents on BMI. Notably, the current literature on BMI antecedents focuses on start-ups [80,168], mature firms [57,61], and social enterprises [53,78], ignoring family businesses. In our review, only three papers focused on BMI in family firms [31,103,136]. These three studies in the literature have shown BMI in family firms to be a challenging and complex task. Therefore, future research should explore in greater depth the specific journeys of family firms in terms of BMI to understand the factors of their success or failure. This will provide valuable insights for family business innovation and sustainability.

Second, BMI is a complex and iterative process that involves companies in different stages of innovative value creation, delivery, and capture mechanisms. Therefore, we believe that dedicated teams should be established to deal with BMI in companies [169]. However, we do not recommend assigning this responsibility solely to the CEO, as they may be negatively affected by factors such as information overload, industry limitations, and the management's efforts to protect its rights by withholding valid information from the CEO. This may compromise the CEO's judgment of BMI [88]. Additionally, while the learning process perspective provides some insights, it does not fully address fundamental questions about the origins of learning experiment scenarios and how experiments are controlled. Future research needs to explore these areas to provide a more comprehensive theoretical foundation for the BMI process. Although existing studies have emphasized the role of organizational capabilities, such as DCs, in the BMI process, none of these studies have analyzed in detail the differences in the capabilities and resources required at different stages of BMI. Therefore, future research should explore the specific capabilities and resources required at each stage of the BMI process and the various barriers that may be encountered, as well as the operational resources and capabilities required to remove these barriers. On the other hand, while some studies have suggested that the BMI process can also focus on changes in internal components [83,88] or adopt a systems perspective [86,87], the interrelationships between internal components and their impact on the BMI process have not been fully explored. Further studies in the literature have shown that there is a large variation in BMI measurement. Although many researchers have conducted empirical studies and measured BMI using the scale developed by Ref. [170], this scale only validates the applicability of BMI theory at the new firm level and cannot take into account new situations that arise in the market and industry. Therefore, we strongly recommend the development of a more advanced and comprehensive measurement instrument to validate the BMI theory and facilitate its further development. Finally, we encourage future BMI process research to focus on the similarities and differences in BMI processes in different contexts and to determine whether the pathways to BMI are different or similar for various types of companies. This will help us gain a deeper understanding of the BMI process and develop more effective strategies for successful BMI implementation.

Third, the study of BMI outcomes is a challenging area that requires further in-depth research and exploration. While existing studies highlight the positive impacts of BMI from different perspectives and approaches, such as its ability to increase productivity, reduce costs, promote economic growth [127], bring about product and service innovation [38], drive sustainable development [166,167], and foster inclusive growth [163], there is still a lack of investigation into how companies that effectively implement BMI prevent imitation and copying by competitors. Achieving BMI outcomes requires considering not only the BM itself but also other key factors such as organizational culture and employee engagement, which have not been fully explored in existing studies. Furthermore, our study found that the role of BMI in internationalization has been overlooked. Thus, future research should comprehensively consider the above factors and explore their impact on BMI outcomes as well as delve into the role of BMI in internationalization to better understand the possible outcomes of BMI. Finally, it is important to note that the primary goal of BMI is to create value and achieve high economic efficiency for the firm. However, the current literature lacks a comprehensive and systematic assessment of BMI outcome indicators and is limited to purely economic criteria [8]. To overcome these limitations, more robust evaluation criteria, such as eco-efficiency, social responsibility, and innovation capacity, are needed in the future to more comprehensively assess the impact of BMI on business and society.

## 7 Conclusion

This study presents a systematic review of the literature on BMI, exploring its antecedents, processes, barriers, and outcomes, while considering the differences in the understanding of BMI across disciplines. As BMI has gained increasing attention from the academic community, we propose a theoretical framework that clarifies the logic of BMI and provides scholars and practitioners with a comprehensive understanding of the current state of research and theoretical development. Additionally, we suggest a set of issues to consider from each perspective, along with a future research agenda based on the identified theoretical framework.

This systematic review provides valuable contributions to both BMI research and management practice. By reviewing the theoretical framework and three research streams, we promote a common understanding of BMI and offer guidance for future BMI research. We found that knowledge about BMI is scattered across various research journals and that the field remains highly heterogeneous, which supports [171] view that BMI is in an isolated state. The empirical studies on BMI have been limited in scope and content, which suggests that future research should use more empirical studies to validate the previous findings [8,10,13,172]. Our integrated theoretical framework is an update and extension of the previous frameworks [8], including a sustainability perspective that had been overlooked in prior literature.

From a management perspective, our study can contribute to refining the theory of BMI, ultimately resulting in more successful implementation in practice. Specifically, through this study, managers can gain a better understanding of the causes and consequences of BMI and avoid a “one-size-fits-all” approach to the BMI process. The success of BMI cannot be attributed to a single factor, but instead involves iterative and recursive phases that require managing multiple BMs simultaneously while switching back and forth between old and new models. As we have developed a framework that summarizes the antecedents, processes, outcomes, types, and barriers that may arise during BMI over the past 20 years, managers can gain a comprehensive understanding of the impact that different factors may have on the process. This knowledge will enable them to respond flexibly to the situations that may arise during the BMI process based on the anticipated impact. In addition, The transparency of our research process allows scholars and practitioners to quickly navigate the literature and understand how the theory of BMI is developing. In summary, our study provides valuable insights into the BMI literature, which can help managers to refine their strategies and ultimately lead to more successful implementation in practice.

While an SLR is an effective method for understanding the state of development of the existing literature on BMI, there are limitations to this approach. For example, our search string was limited to articles on BMI, and related areas, such as sustainable BMI and social BMI, were not included. Additionally, we only evaluated the most cited and influential peer-reviewed publications in English, potentially missing useful ideas from the excluded books and articles. Owing to the high growth stage of the literature on BMI, some recent papers might not have been considered in this review. Finally, as research on BMI is still in its early stages, there is only few extant studies examine its antecedents, barriers, processes, types, and moderating and mediating variables.

## Author contribution statement

All authors listed have significantly contributed to the development and the writing of this article.

## Date availability statement

Date included in article/ referenced in article.

## Additional information

No additional information is available for this paper.

## Declaration of competing interest

The authors declare that they have no known competing financial interests or personal relationships that could have appeared to influence the work reported in this paper.
